# Incidence of Anxiety Diagnosis up to Four Years Post SARS-CoV-2 Infection in the Montefiore Medical Center in the Bronx and New York

**DOI:** 10.3390/diagnostics15202605

**Published:** 2025-10-16

**Authors:** Sagar Changela, Roham Hadidchi, Sophia Wu, Ekram Ali, Alex Liu, Thomas Peng, Tim Q. Duong

**Affiliations:** Department of Radiology, Albert Einstein College of Medicine and Montefiore Health System, Bronx, NY 10461, USA

**Keywords:** anxiety, pandemic, COVID-19, SARS-CoV-2

## Abstract

**Background/Objectives:** Emerging evidence suggests that individuals infected with SARS-CoV-2 are at an elevated risk of developing anxiety. This study investigated the association and risk factors of anxiety diagnosis in COVID-19 patients compared to non-COVID-19 patients up to four years post index date. **Methods:** We conducted a retrospective case–control study on a cohort consisting of 1.3 million patients, of which 85,229 had a clinical anxiety diagnosis (defined by ICD code) and 1,214,598 did not from the Montefiore Medical Center in the Bronx, New York, from 1 March 2020 to 31 January 2024. COVID-19 patients were those who tested positive for SARS-CoV-2 by polymerase chain reaction. COVID-19 negative patients were those who did not have a recorded positive test. The outcome was an anxiety diagnosis between one month and four years post-index date. Analysis was performed with unmatched and matched cohorts. The matched variables included age, sex, race, and ethnicity. The multivariate logistic regression adjusted odds ratio (aOR) and 95% confidence interval were computed. **Results:** COVID-19 was more prevalent among patients with anxiety compared to those without anxiety (6.92% vs. 4.14%, *p* < 0.001). COVID-19 patients were more likely to develop anxiety compared to non-COVID-19 patients (multivariate aOR = 2.13 [2.06–2.20] for unmatched cohort, and aOR = 1.26 [1.22, 1.31] for the matched cohort). Female (aOR = 1.54 [1.52, 1.56]), Black (aOR = 1.38 [1.35, 1.41]), Asian (aOR = 1.48 [1.41, 1.55]) (vs. White), and Hispanic patients were more likely to develop anxiety compared to their counterparts (unmatched cohort). **Conclusions:** COVID-19 is associated with a greater likelihood of having anxiety. Black and Hispanic patients were at higher risk, suggestive of the COVID-19 pandemic exacerbating health disparities. An improved understanding of long-term anxiety is crucial for developing effective interventions and support systems for COVID-19 survivors.

## 1. Introduction

Coronavirus disease of 2019 (COVID-19) [1,2], caused by the severe acute respiratory syndrome coronavirus 2 (SARS-CoV-2), has had profound effects on global public health, extending far beyond the acute physical illness associated with the virus [3,4]. Long COVID, which includes a constellation of symptoms lasting for weeks or months post SARS-CoV-2 infection, is emerging as a critical factor in the sustained mental health burden of the pandemic [5], and emerging evidence suggests that individuals infected with SARS-CoV-2 are at an elevated risk of developing anxiety disorders [3,4].

A meta-analysis by Necho et al., which reviewed studies involving 17,438 COVID-19 patients globally, reporting a pooled anxiety prevalence of 31.9% [6]. A review by Pashazadeh Kan et al. found a higher prevalence of anxiety among COVID-19 as compared with COVID-unexposed patients [7]. Several studies have identified specific factors contributing to anxiety in COVID-19 patients. Clinical factors, such as the severity of infection, symptoms like dyspnea, and intensive care unit admission, are strongly linked to post-COVID anxiety [8]. Psychosocial factors, including social isolation during quarantine, fear of infecting loved ones, and the stigma associated with the disease, further exacerbated anxiety [9,10]. The fear of death and severe illness, and the uncertainty regarding treatment efficacy also played a central role [11]. Vulnerable populations, such as the elderly, have been disproportionately affected due to their increased vulnerability and social isolation [12]. Pregnant women with COVID-19 experience heightened anxiety due to concerns about fetal health, restricted access to prenatal care, and fear of vertical transmission [13]. Healthcare workers infected with SARS-CoV-2 also report significant anxiety, driven by occupational stress, stigma, and fear of reinfection [14].

The neurotropic properties of SARS-CoV-2 also suggest potential direct effects on the central nervous system [15]. Direct neuroinvasion may lead to neuroinflammation or the disruption of neurotransmitter systems and the neural circuits that regulate mood and anxiety, thereby predisposing individuals to anxiety disorders [16]. Furthermore, the systemic inflammation associated with severe COVID-19 can contribute to neuropsychiatric symptoms, including anxiety, through the activation of the hypothalamic–pituitary–adrenal axis and the subsequent dysregulation of stress hormones [17].

Beyond the direct effects of the virus, the COVID-19 pandemic has created an environment conducive to widespread psychological distress, contributing to a rise in anxiety at the population level. The pandemic has introduced multiple stressors, including the threat of infection, prolonged isolation due to lockdowns, economic uncertainty, and disruptions to social support systems [18]. These factors have exacerbated pre-existing mental health conditions and have led to the development of more cases of anxiety, particularly among vulnerable subsets of the population [19]. The constant exposure to pandemic-related information through media and the uncertainty surrounding the trajectory of the pandemic have further fueled anxiety and fear [20].

Despite extensive research into anxiety among COVID-19 patients, several critical gaps remain. Existing studies often focus on short-term post-COVID anxiety, with the long-term risk of anxiety and its persistence not well characterized. The prevalence and specific risk factors for post-COVID anxiety in diverse urban populations remain underexplored.

The goal of this study was to investigate the long-term risk of clinical anxiety diagnosis and identify risk factors of clinical anxiety diagnosis in COVID-19 patients compared to non-COVID-19 patients over a period of up to four years post index date. The data were derived from a large urban, diverse population in the Bronx, New York (U.S.A), an epicenter of the early pandemic and subsequent surges of infection. Our analysis provides a glimpse of the long-term risk of anxiety among COVID-19 patients.

## 2. Methods

### 2.1. Data Sources

Due to the retrospective nature of the study, the need for obtaining informed consent was waived by the Einstein-Montefiore Institutional Review Board, and all protocols in this study were approved by the Einstein-Montefiore Institutional Review Board (#2021-13658). All methodology was in accordance with the ethical standards laid down in the 1964 Declaration of Helsinki and its later amendments [21]. We derived data from the Montefiore Medical Center in the Bronx in New York City (U.S.A) from 1 March 2020 to 12 January 2024. The health system consists of multiple hospitals and dozens of clinics that serve a racially and ethnically diverse population in an urban setting. The Bronx was an early epicenter of the COVID-19 pandemic and subsequent surges. Data were extracted as described previously [22,23,24,25,26,27,28,29,30,31,32]. Deidentified clinical data were harmonized to the OMOP Common Data Model (CDM) version 6, which maps heterogeneous sources to standardized vocabularies, enabling consistent analyses across electronic health records, claims data, and coding systems such as ICD-10 [33]. We used ATLAS (OHDSI) to browse concepts and define cohorts in the CDM environment. Cohort extracts were then exported to SQLite and queried with DB Browser for SQLite (v3.12.2). To assure data quality, we cross-checked key variables via manual chart review in sampled patients.

### 2.2. Study Population

We selected patients who had a clinical anxiety diagnosis made by healthcare professionals (as documented by ICD-10 diagnostic codes) and those who did not have an anxiety diagnosis (controls) after March 2020. COVID-19 positive patients were those with a recorded positive test for SARS-CoV-2 by polymerase chain reaction (PCR). COVID-19 negative patients were those who did not have a recorded positive SARS-CoV-2 PCR test (either never tested or always tested negative when tested). ICD-10 codes and OMOP concept IDs for anxiety are included in Supplementary Table S1.

### 2.3. Variables

Demographic characteristics including age at index encounter, race, ethnicity, and sex were tabulated. Race and ethnicity were based on patient self-identification. Pre-existing comorbidities, including hypertension (HTN), chronic kidney disease (CKD), diabetes, cardiovascular diseases (CVD, which included congestive heart failure, myocardial infarction, and coronary artery disease), chronic obstructive pulmonary disease (COPD), asthma, obesity, and anxiety, were identified based on ICD-10 codes. Anxiety was the outcome and COVID-19 status was the primary predictor.

### 2.4. Statistical Analysis

The Sklearn, Statsmodel, SciPy, and lifeline libraries in Python (version 3.8.19) were used to conduct the statistical analyses. The Pearson χ2 or Fisher’s exact tests were used to compare categorical variables for patient demographic characteristics and comorbidities, and the unpaired two-tail *t*-test was used for continuous variables. Crude and adjusted logistic regression models were used to estimate the odds ratio (OR) and 95% confidence interval (CI) on unmatched data. Data were matched 1:2 on age, sex, race, and ethnicity using the propensity score matching, and a similar analysis was performed on the matched cohort.

## 3. Results

**Figure 1:**
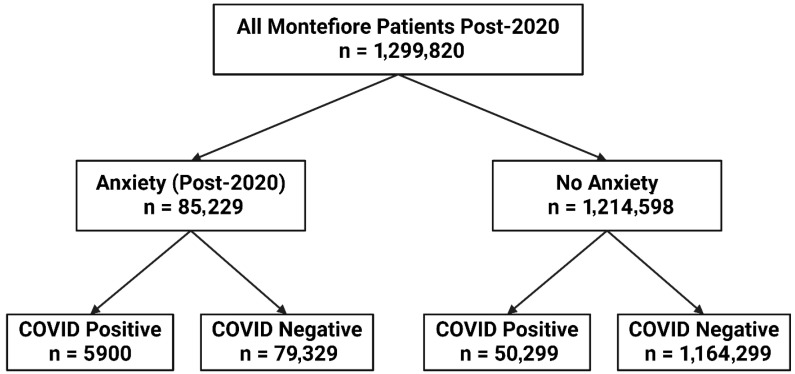
Patient selection flowchart for case–control study design.

Figure 1 shows the flowchart of patient selection. In a cohort of 1.3 million patients from 1 March 2020 to 12 January 2024, 85,229 had an anxiety diagnosis and 1,214,598 did not. Among those with anxiety, 5900 were COVID-19 positive and 79,329 were not. Among those without anxiety, 50,299 were COVID-19 positive and 1,164,299 were not.

### 3.1. Unmatched Data

Table 1 presents the demographic and clinical profiles of patients with and without anxiety (unmatched cohort). COVID-19 was significantly more prevalent among patients with anxiety compared to those without (6.92% vs. 4.14%, *p* < 0.001). Patients with anxiety had a similar age (43.44 ± 21.43 vs. 38.23 ± 24.28, *p* = 0.87), with a higher proportion of female patients (69.21% vs. 54.50%, *p* = 0.001), higher proportion of Black patients (26.11% vs. 25.01%, *p* = 0.001), and higher proportion of Hispanic patients (46.61% vs. 32.70%, *p* = 0.001) compared to those without anxiety. Pre-existing comorbidities were more prevalent among patients with anxiety, such as HTN (31.17% vs. 15.26%, *p* < 0.001) and diabetes (15.89% vs. 7.85%, *p* < 0.001).

Table 2 shows the logistic regression model results in the unmatched cohort. The crude model showed that the odds of having anxiety were 4.56 [95% CI, 4.44–4.69] times higher among COVID-19 positive patients compared to COVID-19 negative patients. Age, sex, and other comorbidities were also significantly associated with developing anxiety. After accounting for these covariates, the COVID-19 adjusted OR was 2.13 [2.06–2.20]. Female (aOR = 1.54 [1.52, 1.56]), Black (aOR = 1.38 [1.35, 1.41]), Asian (aOR = 1.48 [1.41, 1.55]) (vs. White), and Hispanic patients were more likely to develop anxiety compared to their counterparts (unmatched cohort).

### 3.2. Matched Data

Table 3 shows the patient profile of the matched cohort. The matched cohort consisted of 85,229 anxiety patients and 170,458 controls. All comorbidities were significantly higher among the anxiety patients compared to control patients (*p* < 0.05), except for CKD. Adjusted model controlling for comorbidities and pre-existing anxiety showed that the odds of having anxiety was 1.26 [1.22,1.31] times higher among COVID-19 positive patients compared to COVID-19 negative patients.

## 4. Discussion

This study investigated the association of clinical anxiety diagnosis and SARS-CoV-2 infection up to four years post SARS-CoV-2 infection in a large diverse population in the Bronx, an epicenter of the early COVID-19 pandemic and subsequent surges of infections. Individuals with a history of COVID-19 were more likely to develop anxiety compared to those without a history of COVID-19 after adjusting for confounders (aOR = 2.13 [95% CI 2.06–2.20] in the unmatched cohort, and aOR = 1.26 [95% CI 1.22, 1.31] in matched cohort). Black, Asian, and Hispanic patients had higher odds compared to their counterparts.

### 4.1. Demographic Factors

In the unmatched cohort, individuals who had COVID-19 were more likely to develop anxiety compared to those who did not after adjusting for confounders (aOR = 2.13 [95% CI 2.06–2.20]). Older age was associated with higher odds of anxiety. Black and Hispanic patients also had greater odds than White and non-Hispanic patients, respectively.

COVID-19 has had an outsized impact on marginalized communities [34,35]. Restricted access to care, greater underlying illness, overcrowded living environments, and essential high-exposure work collectively heightened COVID-19’s burden in vulnerable populations [36,37]. Evidence from multiple studies indicates that socioeconomic disadvantages or unmet health-related social needs are associated with increased SARS-CoV-2 infection and poorer acute outcomes [38,39,40,41,42,43].

Other contributors may have further amplified COVID-19-associated health inequities. Vulnerable groups—including healthcare workers, other frontline employees, and marginalized communities—have shown elevated anxiety levels, partly attributable to COVID-19’s direct and indirect effects [44,45]. These populations faced a higher risk of infection, limited access to mental health services, and heightened exposure to pandemic-related stressors, contributing to the overall burden of anxiety [46,47].

### 4.2. Comorbidity Factors

Our findings also revealed significant associations between various comorbid conditions and the likelihood of anxiety, underscoring the complex interplay between chronic illnesses and mental health. Conditions such as HTN, obesity, COPD, and asthma were found to be associated with higher odds of anxiety. The increased prevalence of anxiety in patients with HTN, obesity, COPD, and asthma may be attributed to several overlapping physiological and psychosocial mechanisms. Chronic diseases often involve systemic inflammation, autonomic dysfunction, and alterations in hypothalamic–pituitary–adrenal axis activity, all of which have been implicated in the pathophysiology of anxiety [48,49]. Furthermore, the daily burden of managing these conditions, coupled with the fear of disease progression, likely exacerbates psychological stress and contributes to anxiety. The slightly lower odds of anxiety observed in patients with DM and CKD may seem counterintuitive, as these are conditions associated with significant morbidity. It is possible there could be interactions among comorbidities (i.e., patients who had HTN might also have DM) which could result in unexpected ORs. Further investigation is needed.

Not surprisingly, pre-existing anxiety emerged as the strongest predictor of subsequent anxiety. This finding underscores the chronic and often recurrent nature of anxiety disorders. Patients with a history of anxiety may have a heightened susceptibility to new or exacerbated symptoms in response to stressors, including the diagnosis or management of chronic illnesses. This highlights the importance of the early identification and sustained management of anxiety, especially in individuals with predisposing factors or comorbid conditions.

### 4.3. Matched Cohort

It is possible that demographics could confound outcomes despite multivariate analysis. We thus performed a multivariate analysis with matching for age, sex, race, and ethnicity. The multivariate analysis of matched data showed similar odds for anxiety (aOR = 1.26 [95% CI: 1.22–1.31]). The slightly lower COVID-19 aOR after matching may suggest that some of the matched confounders may have interacted with the risk of anxiety.

The association between COVID-19 and anxiety may be reflective of biological processes triggered by the infection, psychological stress from illness and isolation, and broader societal disruptions caused by the pandemic [50,51]. One of the primary avenues through which SARS-CoV-2 may contribute to anxiety is its impact on the central nervous system. The virus can cause neuroinflammation and disrupt neural circuits involved in mood regulation, leading to increased anxiety [52]. Evidence has shown that cytokine storms, a hallmark of severe COVID-19 cases, may alter brain function by increasing pro-inflammatory cytokine levels [53]. These inflammatory responses have been linked to the onset of mood disorders, including anxiety [54,55]. In addition to neuroinflammation, the infection may affect neurotransmitter systems that regulate anxiety [56,57]. For instance, alterations in the serotonergic and dopaminergic pathways, both of which are known to influence anxiety, have been observed in COVID-19 [58,59,60]. The direct and indirect effects of the virus on neurotransmitter balance may thus contribute to the heightened incidence of anxiety among those infected.

The pandemic has also brought about several psychological stressors, especially for individuals diagnosed with COVID-19. These include the fear of severe illness or death, uncertainty regarding prognosis [9,10,11], and the potential for long-term complications, including long COVID, which can include persistent symptoms such as fatigue, cognitive dysfunction, and dyspnea. Long COVID is also associated with anxiety, possibly due to its prolonged and unpredictable course [61,62]. Moreover, the requirement for isolation and quarantine during infection may lead to loneliness and loss of social support, both of which are risk factors for anxiety disorders [63]. Social isolation, in combination with health-related anxiety, may exacerbate pre-existing mental health vulnerabilities or trigger new-onset anxiety [64]. Among individuals who had no prior history of mental illness, some studies have reported new diagnoses of anxiety following a SARS-CoV-2 infection [65,66].

In the broader context, societal responses to the COVID-19 pandemic, including lockdowns, economic insecurity, and the disruption of daily life, have contributed to a global increase in anxiety levels [67,68,69]. For individuals infected with SARS-CoV-2, these stressors are compounded by the physiological burden of the disease [52]. A meta-analysis of the global mental health impact of COVID-19 reported a significant rise in anxiety, especially in populations experiencing severe outbreaks and lockdowns [67].

### 4.4. Strengths and Limitations

Our study offers multiple strengths. This analysis had one of the longest study periods, extending up to January 2024. The large, Bronx-based cohort, significantly affected during the initial COVID-19 waves, enables an examination of potential long-term anxiety effects. Our data consisted of a diverse urban population, with large proportions of Hispanic and Black patients. Additionally, a clinical diagnosis of anxiety was employed as opposed to questionnaire survey data. There are several limitations. Our analyses include only patients who returned to our health system, which may bias the sample toward individuals with more severe illness. However, electronic medical records included patients returning for any reason (e.g., routine office visits). The misclassification of anxiety diagnoses is also possible. Nevertheless, misclassification likely occurred similarly across COVID-19 positive and negative groups, so our conclusions should remain unaffected. In our study, we only counted patients who were seen for anxiety after the index date. We did not differentiate whether patients with prior anxiety could be more susceptible post-COVID-19, nor did we determine whether post-index anxiety diagnoses represented a new onset or recurrence of a chronic condition. We purposely did not exclude patients with pre-existing anxiety, as we were also interested in examining the burden of anxiety after the index date in this subgroup. Our study was not intended to investigate the causality between COVID-19 and anxiety, which is an important question for future research. A misclassification of COVID-19 status could occur because patients could have been tested elsewhere and not documented in our electronic medical records. It is possible to use only patients who tested negative for COVID-19 as controls, but doing so would not avoid the future misclassification of COVID-19 status because patients could have been tested elsewhere later. As majority of patients were COVID-19 negative, we feel confident that our conclusion using patients without a positive test is valid. Anxiety diagnoses across time could be influenced by factors such as vaccination availability and virulence of the virus strains across the pandemic. Vaccine status was not consistently captured for patients vaccinated elsewhere in most health systems including ours, so it was not evaluated as a predictor of the outcome. Moreover, the pandemic also interrupted health services due to the lockdown and avoidance of health facilities, which could significantly confound any temporal analysis associated with vaccination availability and virulence of the virus strain across the pandemic. The temporal sequence between exposure and outcome is another limitation, as the case–control study design limits our ability to infer causality. Furthermore, the study sample may not fully represent the broader population, which may affect the external validity of our findings. Further studies are needed to assess causality. Future studies will need to include longer follow-ups and larger patient cohorts. As with any retrospective study, there could be other unintended patient selection biases and latent confounds.

## 5. Conclusions

In a large diverse population in the Bronx, we found that individuals with a history of COVID-19 were more likely to develop anxiety compared to those without a history of COVID-19 after adjusting for confounders. Black, Asian, and Hispanic patients had higher odds compared to their counterparts. An improved understanding of the relationship between COVID-19 and anxiety disorders is crucial for developing effective interventions and support systems for individuals affected by COVID-19.

## Figures and Tables

**Table 1:** Profiles of patients with and without anxiety (unmatched). SD, standard deviation; COPD, chronic obstructive pulmonary disease.

	Anxiety (n = 85,229)	No Anxiety (n = 1,214,598)	*p* Value
COVID-19	5900 (6.92%)	50,299 (4.14%)	**<0.0001**
Age, mean ± SD (Years Old)	43.44 ± 21.43	38.23 ± 24.28	0.87
Female, n (%)	58,983 (69.21%)	661,944 (54.50%)	**<0.0001**
**Race/Ethnicity**			
White	14,182 (16.64%)	160,737 (13.23%)	**<0.0001**
Asian	2231 (2.62%)	34,892 (2.87%)	**0.00002**
Black	22,251 (26.11%)	303,792 (25.01%)	**<0.0001**
Other	46,552 (54.62%)	703,707 (57.94%)	**<0.0001**
Hispanic	39,724 (46.61%)	397,156 (32.70%)	**<0.0001**
**Pre-Existing Comorbidities**			
Hypertension	26,570 (31.17%)	185,297 (15.26%)	**<0.0001**
Chronic Kidney Disease	6779 (7.95%)	42,958 (3.54%)	**<0.0001**
Diabetes	13,544 (15.89%)	95,315 (7.85%)	**<0.0001**
Cardiovascular Diseases	4682 (5.49%)	29,803 (2.45%)	**<0.0001**
COPD	3166 (3.71%)	11,715 (0.96%)	**<0.0001**
Asthma	16,601 (19.48%)	81,834 (6.74%)	**<0.0001**
Obesity	21,049 (24.70%)	108,852 (8.96%)	**<0.0001**
Pre-Existing Anxiety	28,523 (33.47%)	35,647 (2.93%)	**<0.0001**

**Table 2:** Crude and multivariable odds ratios (ORs) (unmatched data). CI, confidence interval; COPD, chronic obstructive pulmonary disease. * indicates significance.

Variable	Crude OR	95% CI	Adjusted OR	95% CI
COVID-19	4.56 *	(4.44, 4.69)	2.13 *	(2.06, 2.20)
Age (Years Old)	1.01 *	(1.008, 1.009)	1.00	(1.00, 1.002)
Female vs. Male	1.89 *	(1.85, 1.89)	1.54 *	(1.52, 1.56)
Black vs. White	1.06 *	(1.04, 1.08)	1.38 *	(1.35, 1.41)
Asian vs. White	0.91 *	(0.87, 0.95)	1.48 *	(1.41, 1.55)
Other vs. White	1.00	(0.60, 1.67)	1.01	(0.57, 1.78)
Hispanic vs. Non-Hispanic	1.80 *	(1.77, 1.82)	1.79 *	(1.76, 1.83)
Hypertension	2.52 *	(2.48, 2.56)	1.25 *	(1.22, 1.28)
Chronic Kidney Disease	2.36 *	(2.30, 2.42)	1.05 *	(1.01, 1.09)
Diabetes	2.22 *	(2.18, 2.26)	0.97 *	(0.94, 0.99)
Obesity	3.33 *	(3.28, 3.39)	1.58 *	(1.54, 1.61)
Pre-Existing Anxiety	16.64 *	(16.35, 16.94)	11.26 *	(11.05, 11.47)
Cardiovascular Diseases	2.31 *	(2.24, 2.39)	0.89 *	(0.83, 0.96)
COPD	3.97 *	(3.81, 4.13)	1.1 *	(1.05, 1.16)
Asthma	3.35 *	(3.29, 3.41)	1.56 *	(1.53, 1.60)

**Table 3:** Profiles of patients with and without anxiety who returned to our health system (matched). Matching was performed 1:2 on age, sex, race, and ethnicity. SD, standard deviation; COPD, chronic obstructive pulmonary disease.

	Anxiety (n = 85,229)	No Anxiety (n = 170,458)	*p* Value
COVID-19	5900 (6.92%)	7843 (4.60%)	**<0.0001**
Age, mean ± SD (Years Old)	43.44 ± 21.43	43.29 ± 21.88	0.99
Female, n (%)	58,983 (69.21%)	117,865 (69.15%)	0.99
**Race/Ethnicity**			
White	14,182 (16.64%)	28,364 (16.64%)	1.00
Asian	2231 (2.62%)	4462 (2.62%)	1.00
Black	22,251 (26.11%)	44,502 (26.11%)	1.00
Other	46,552 (54.62%)	91,269 (53.54%)	1.00
Hispanic	39,724 (46.61%)	64,113 (37.61%)	0.99
**Pre-Existing Comorbidities**			
Hypertension	26,570 (31.17%)	30,793 (18.06%)	**<0.0001**
Chronic Kidney Disease	6779 (7.95%)	6709 (3.94%)	0.89
Diabetes	13,544 (15.89%)	15,603 (9.15%)	**<0.0001**
Cardiovascular Diseases	4682 (5.49%)	4673 (2.74%)	**<0.0001**
COPD	3166 (3.71%)	1916 (1.12%)	**<0.0001**
Asthma	16,601 (19.48%)	12,323 (7.23%)	**<0.0001**
Obesity	21,049 (24.70%)	18,536 (10.87%)	**<0.0001**
Pre-Existing Anxiety	28,523 (33.47%)	6295 (3.69%)	**<0.0001**

## Data Availability

Further inquiries can be directed to the corresponding author.

## Supplementary Materials

The following supporting information can be downloaded at https://www.mdpi.com/article/10.3390/diagnostics15202605/s1. Supplementary Table S1. Pre-set search terms used to define anxiety disorders and other conditions.
